# Human-Friendly Light-Emitting Diode Source Stimulates Broiler Growth

**DOI:** 10.1371/journal.pone.0135330

**Published:** 2015-08-13

**Authors:** Jinming Pan, Yefeng Yang, Bo Yang, Wenhua Dai, Yonghua Yu

**Affiliations:** 1 College of Biosystems Engineering and Food Science, Zhejiang University, Hangzhou, 310058, China; 2 Agriculture Machinery Technique Extending Station, Jiaxing, 31400, China; University of Hong Kong, CHINA

## Abstract

Previous study and our laboratory have reported that short-wavelength (blue and green) light and combination stimulate broiler growth. However, short-wavelength stimuli could have negative effects on poultry husbandry workers. The present study was conducted to evaluate the effects of human-friendly yellow LED light, which is acceptable to humans and close to green light, on broiler growth. We also aimed to investigate the potential quantitative relationship between the wavelengths of light used for artificial illumination and growth parameters in broilers. After hatching, 360 female chicks (“*Meihuang*” were evenly divided into six lighting treatment groups: white LED strips (400–700 nm, WL); red LED strips (620 nm, RL); yellow LED strips (580 nm, YL); green LED strips (514 nm, GL); blue LED strips (455 nm, BL); and fluorescent strips (400–700 nm, FL). From 30 to 72 days of age, broilers reared under YL and GL were heavier than broilers treated with FL (*P* < 0.05). Broilers reared under YL obtained the similar growth parameters with the broilers reared under GL and BL (*P* > 0.05). Moreover, YL significantly improved feeding efficiency when compared with GL and BL at 45 and 60 days of age (*P* < 0.05). In addition, we found an age-dependent effect of light spectra on broiler growth and a quantitative relationship between LED light spectra (455 to 620 nm) and the live body weights of broilers. The wavelength of light (455 to 620 nm) was found to be negatively related (*R* ^*2*^ = 0.876) to live body weight at an early stage of development, whereas the wavelength of light (455 to 620 nm) was found to be positively correlated with live body weight (*R* ^*2*^ = 0.925) in older chickens. Our results demonstrated that human-friendly yellow LED light (YL), which is friendly to the human, can be applied to the broilers production.

## Introduction

Colored artificial light has been reported to affect metabolic activity and improve productive performance in poultry for many years. Incandescent lamps [1], fluorescent lamps [2], ultraviolet (UV) light lamps [3], commercial colored lamps [4] and filtered light [5] have been successfully used in poultry production. However, the results of studies of the effects of different light spectra on broiler growth have been inconsistent. Although green light has been reported to improve growth [2, 6–8], mature female Japanese quail were found to have lower body weights when reared under green light or blue light compared with those reared under red light or white light emitted from an incandescent lamp [9]. Incandescent pink light was shown to decrease chicken hatching weights, while incandescent white light was found to increase chicken hatching weights [10]. Green light has been found to enhance early chick embryo growth, as did blue [11] and ultraviolet (UV) light [3].

The inconsistencies in these results probably resulted from variability in the light sources [12], intensities [13, 14], and schedules [15] and from differences in the species, sexes and ages of the chickens studied [7–9, 16]. With the development of electronics and semiconductor science, monochromatic light produced by light-emitting diodes (LEDs) has been introduced as a new, alternative form of light that can be used in poultry husbandry and that can be used to eliminate some of these inconsistencies. An investigation using LEDs showed that broilers exposed to green light (560 nm) or blue light (480 nm) from LEDs obtained greater body weights [12] [17]. Previous study in our laboratory showed the combination of the pure green and blue LEDs also can affect the growth and physiology in broilers [18]. However, if green or blue LEDs were used in poultry husbandry, then poultry husbandry workers, who must feed or guard broilers or clean the areas in which broilers are housed, during the entire rearing period (as poultry production in developing countries, including China, is not automatic), may most likely feel very uncomfortable working under such light. It has been reported that short-wavelength light resulted in human health problem [19–26]. Specially, stimulus with blue or green light could potentially have negative effects on poultry husbandry workers, including disrupted circadian rhythms, disrupted patterns of melatonin secretion, disturbed sleep, reduced alertness, and altered cognition and emotional responses [22, 24, 27–31]. However, these effects were not found when subjects were exposed to long-wavelength light [32].

To reduce the potential hazards to poultry workers, human-friendly LED spectra should be introduced into poultry production. Wavelengths in the range of 570–600 nm are perceived as yellow by the human eye, and yellow light is acceptable to the human [33, 34]. It has also been reported that 560 nm green light promotes broiler growth [12, 17]. Thus, yellow LED lights that emits light at a wavelength of 580 nm, which is close to 560 nm and is pleasant to the human eye, were selected for this study, and we evaluated the effect of artificial light using 580-nm yellow LED lights on broiler growth. In addition, the quantitative relationship between lighting spectra and growth performance in broilers is poorly understood. The narrow band of light emitted by LEDs allows us to establish the potential quantitative relationships between specific wavelengths of light used for artificial illumination and growth parameters in broilers more accurately. Thus, the objectives of this study were first to evaluate the effect of human-friendly yellow LED light (580 nm) on broiler growth and second to investigate the potential quantitative relationship between artificial lighting spectra and growth parameters in broilers.

## Materials and Methods

### Animals and housing

A total of 360 broiler chickens (“*Meihuang*”; age 1 day; mean body weight 30.5 g) were purchased from a commercial hatchery (Guangda Breeding Co. Ltd., Hangzhou, Zhejiang Province, China) and used in this study. The genetic performance of this medium-growing broiler strain is very stable, and it is certified by the China Agricultural Ministry as one of the two national gene pools of native broiler libraries [18]. On day 1, all chicks were randomly housed in six light-controlled rooms, with 60 birds per light treatment. Each room was divided into four equal-sized replicate pens, with 15 birds per pen. Each pen had its own independent light system and was covered with fluorescent fabrics to avoid light pollution from other sources. All birds were weighed individually at 30 days of age, and the average body mass was calculated immediately for each treatment. To maintain uniformity, 5 birds (such as heaviest, smallest and lame birds) were eliminated per treatment, and 10 broilers from each replicate without creating a deviation from the original average data (20 birds in two replicates of 10 birds from each light treatment group) were reared until the end of the experiment (72 d of age). All chicks had *ad libitum* access to food and water, and their diets were formulated to meet the nutrient recommendations for poultry (NRC, 1994). The dry bulb temperature and relative humidity were measured once every day, using data loggers (TH602F, Anymetre Co. Ltd., China) to ensure that the temperature and relative humidity conditions were similar in all compartments. The average environmental temperature and relative humidity were 25.3°C and 67.5%, respectively. These conditions were maintained via an electric thermostat and a ventilator throughout the period of the experiment.

### Experimental protocol

Upon arrival, each group of treated broilers was exposed to the following six lighting treatments: (1) a cell (replicate) equipped with two white LED strips (400–700 nm, WL group); (2) a cell (replicate) equipped with two red LED strips (620 nm, RL group); (3) a cell (replicate) equipped with two yellow LED strips (580 nm, YL group); (4) a cell (replicate) equipped with two green LED strips (514 nm, GL group); (5) a cell (replicate) equipped with two blue LED strips (455 nm, BL group); or (6) a cell (replicate) equipped with two fluorescent lamps (400–700 nm, FL group, as a control). Illumination was provided using LED strips (Langtuo Biological Technology Co. Ltd., China). Each group of LEDs was placed 75 cm above the broilers using plastic ties that were attached to the ceiling. The pulse width modulation (PWM) uses a driving current that is determined from the peak current, period of repetition, and pulse duty. The pulse duty is the ratio of ON time to the period that controls the average light intensity. Therefore, in this study, we used the PWM method to control the light intensity precisely and a radiometer (AR823, Digital Lux Meter Co. Ltd., China) to measure the intensity as our previous study [18]. In addition, the intensity was measured in each pen at 5 locations (the four corners and the center of the floor) to maintain a uniform intensity that was the same in each room. Typically, 15 lux is sufficient for the normal growth of chickens [35]. However, young chickens exposed to brighter light can more easily adapt to environments and find food and water. Thus, during the first three days (day 0 to day 3), the light intensity was maintained at a relatively brighter level (30 lux) in all rooms. Following this period, the light intensity was reduced to 15 lux. The lighting schedule was 23L:1D on the first day to allow the broilers adapt to the environment; the light duration was then reduced by 1 hour per day until the lighting schedule reached 16L:8D (on at 08:00 AM, off at 24:00 PM), which was maintained for the remaining days. This study was carried out in strict accordance with the recommendations in the Guide for the Care and Use of Animals of the Zhejiang University. The protocol was approved by the Committee on the Ethics of Animal Experiments of Zhejiang University.

### Growth parameter measurements

Each of the light treated birds had similar initial body weights: 30.4 ± 0.2 (WL), 30.8 ± 0.3 g (RL), 30.2 ± 0.4 (YL), 30.6 ± 0.6 (GL), 30.6 ± 0.5 (BL) and 30.3 ± 0.3 (CFL). For each group, live body weights (g) were recorded at 30, 45, 60 and 72 d of age. Then the body weight gain was calculated. Daily feed consumption (g) was measured. Feed conversion ratio (FCR, g/g) will be calculated by dividing feed consumption with body weight gain and correct for mortality. Feed conversion ratio = (final period body weight − initial period body weight)/period food consumption. At the end of the trial (78 days of age), after being fasted for 12 h, 3 broilers of each replicate, for a total of 72 broilers, were selected randomly, killed by exsanguination, plucked, and eviscerated to measure the absolute weights of the edible viscera (heart, liver and muscle stomach) and drumstick (tibia and thigh). The tibia was measured on the back of the left shank, from the top of the back toe to the top of the shank. The thigh was from the top of the shank to the end of the drumstick. Comb heights (mm) were measured with a vinyl metric measuring tape as an indicator of developmental performance. Non-stressful conditions were provided on the slaughter line, and the birds were slaughtered using a slaughter funnel to prevent wing-flapping and stress during slaughter [17]. All procedures were approved by the Animal Care and Use Committee of Zhejiang University.

### Statistical analysis

Data were analyzed using SPSS Statistical software (V. 20.). A factorial analysis of the data was conducted to analyze the effects of rooms and light treatments. Rooms were found not to have significant effects on any treated variables, and the results were retested via a one-way analysis of variance (ANOVA). ANOVA was performed to analyze the effects of LED strips on the production performance of chickens. Homogeneity of variance was verified for each data set, and no data transformations were applied. When appropriate, post hoc comparisons were made using least significant differences. Data are presented as means ± SEMs. In every case, differences between groups were considered statistically significant if *P* < 0.05. The relationship between light wavelength and the body weight were also analyzed via regression analysis: linear regression models were applied, using SPSS software [36].

## Results

### Live body weights

A significant increase in live body weight was observed in broilers reared under BL as early as 30 days of age compared with the WL and FL groups (*P* < 0.05; Table 1). At 45 days of age, broilers reared under GL had a higher live body weight compared with broilers from the WL and FL groups (*P* < 0.05; Table 1). By 60 days and 72 days of age, YL birds were heavier than those in the WL and FL groups (*P* < 0.05; Table 1). However, at these ages, no difference was found in live body weight between the WL and FL groups (*P* > 0.05). YL, GL and BL increased live body weight in contrast to broad-spectrum light (WL and FL), whereas no difference in live body weight was found between broilers exposed to RL and broilers exposed to WL or FL, suggesting that broiler growth may be suppressed by red light rather than enhanced by yellow, green or blue light (Table 1).

**Table 1:** Live body weights (LBW) and feed conversion ratio (FCR) of broilers reared under different qualities of light. Each group of treated broilers was exposed to either WL (white LED), RL (red LED), YL (yellow LED), GL (green LED), BL (blue LED) or FL (fluorescent lamp). Data are expressed as means ± SEMs (n = 4).

Item	Light treatments groups					
	WL	RL	YL	GL	BL	FL
LBW (g)						
30-d^1^	364.1 ± 2.4^bc^	360.4 ± 5.6^bc^	376.6 ± 6.7^ab^	381.0 ± 2.2^ab^	388.7 ± 6.2^a^	351.1 ± 1.9^c^
45-d	593.9 ± 11.6^ab^	607.0 ± 13.5^ab^	618.4 ± 11.2^ab^	624.8 ± 13.2^a^	617.6 ± 15.6^ab^	585.8 ± 11.3^b^
60-d	841.3 ± 20.1^bc^	885.0 ± 25.6 ^ab^	898.5 ± 29.7^a^	884.2 ± 20.6^ab^	905.8 ± 22.0^a^	839.0 ± 20.5^c^
72-d	1092.6 ± 33.3^c^	1179.1 ± 26.6^a^	1180.3 ± 25.0^a^	1166.4 ± 27.9^ab^	1154.0 ± 29.3^abc^	1103.2 ± 27.4^bc^
FCR^2^ ^(g/g)^						
45-d	2.44 ± 0.10^a^	2.23 ± 0.01^b^	1.98 ± 0.09^c^	2.20 ± 0.04^b^	2.65 ± 0.06^a^	2.59 ± 0.003^a^
60-d	2.76 ± 0.05^b^	2.63 ± 0.06^b^	2.53 ± 0.08^b^	2.49 ± 0.04^c^	2.55 ± 0.08^b^	3.02 ± 0.005^a^
72-d	3.15 ± 0.20^a^	2.73 ± 0.28^a^	2.82 ± 0.09^a^	3.18 ± 0.04^a^	3.31 ± 0.06^a^	3.10 ± 0.02^a^

^1^d indicates day.

^2^FCR = = (final period body weight − initial period body weight)/period food consumption.

^abc^Bars marked with different letters are significantly different from each other (*P* < 0.05).

**Fig 1:**
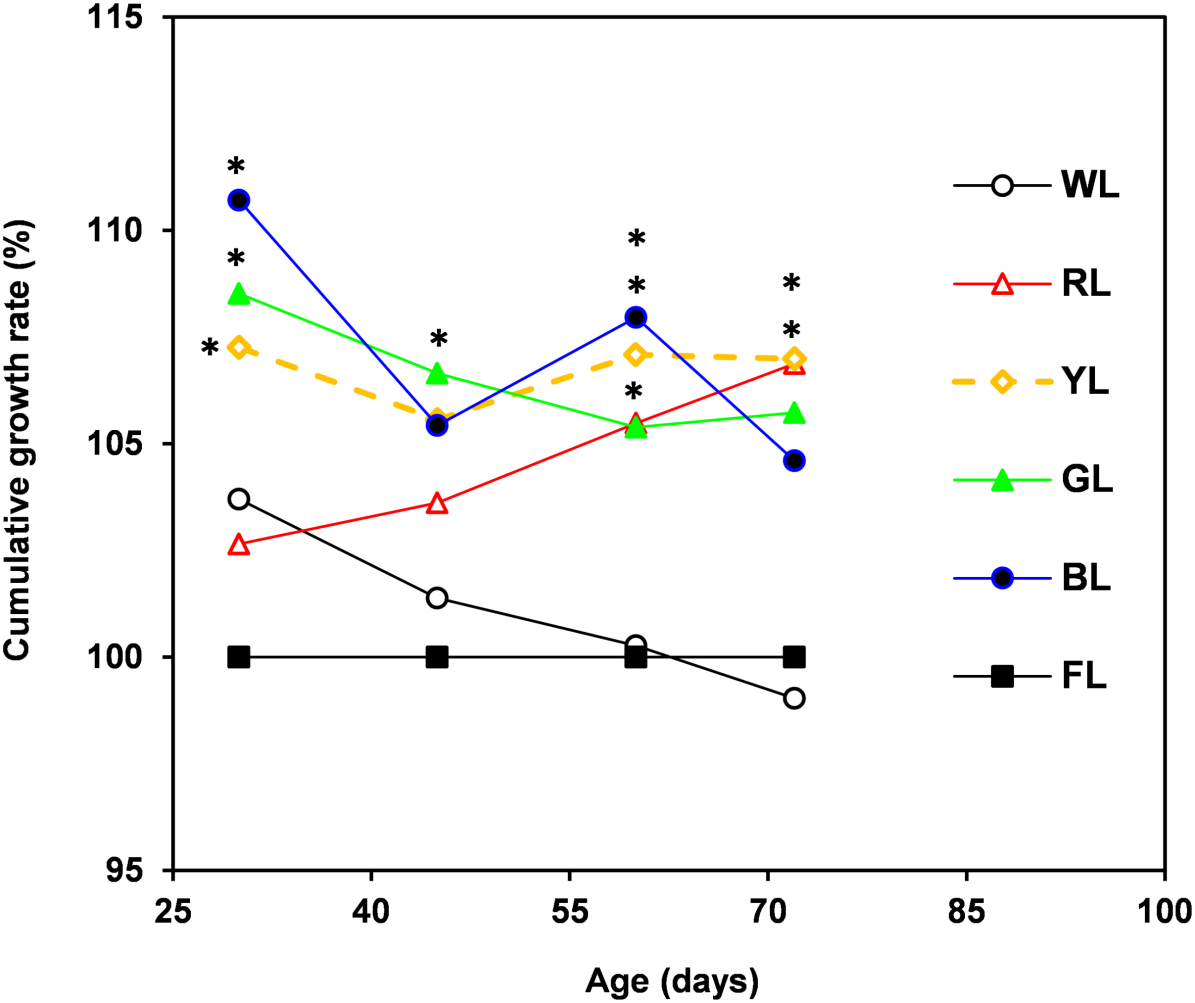
Relative growth rate (% of CFL) of broilers reared under WL (white LED), RL (red LED), YL (yellow LED), GL (green LED), BL (blue LED) or FL (fluorescent lamp).

**Fig 2:**
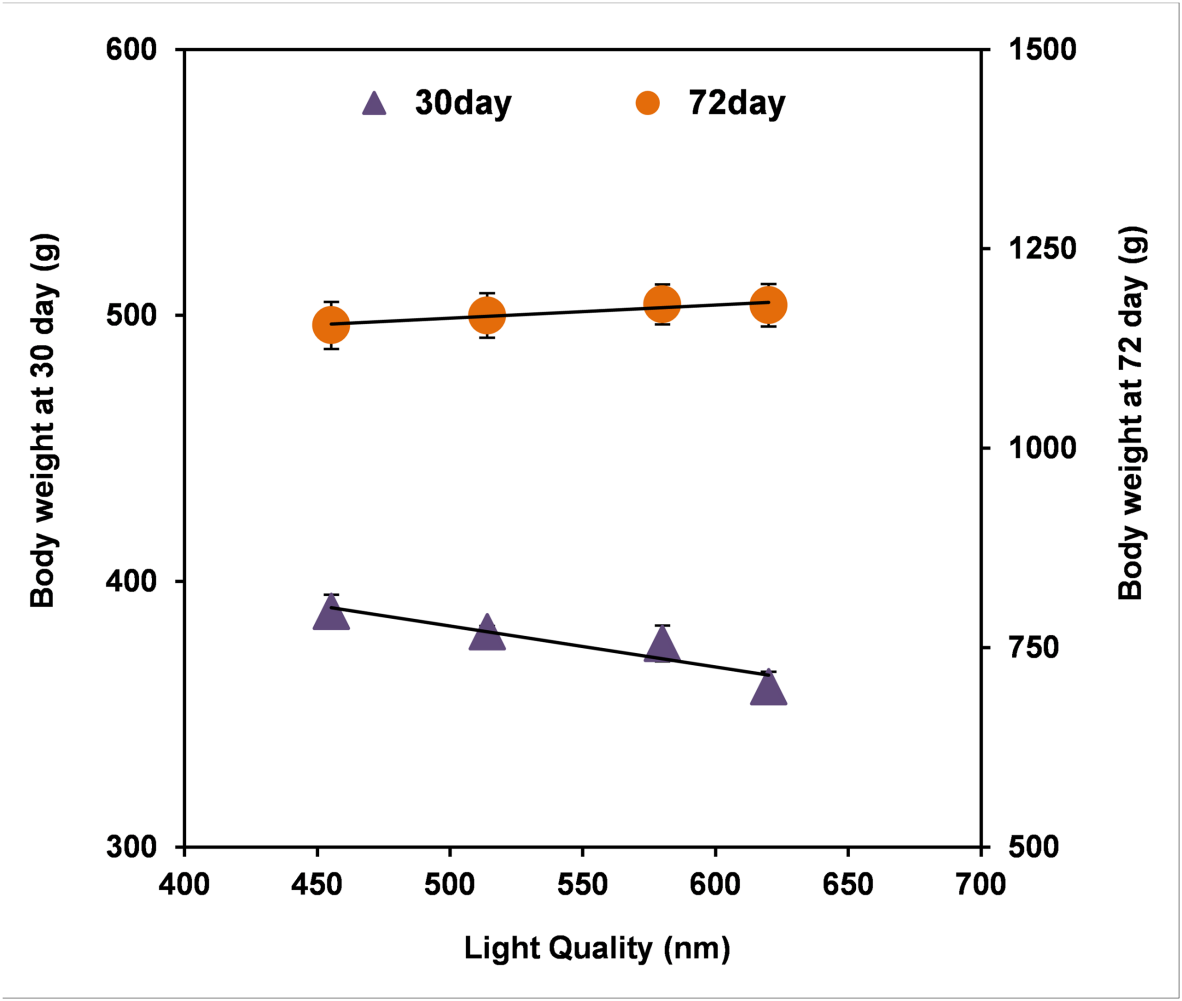
The linear regression of live body weights of broilers on light quality. Broilers were exposed to wavelengths from 455 to 620 nm, either to RL (red LED), YL (yellow LED), GL (green LED) or BL (blue LED). Live body weights at an early age (30 days; triangle) and later age (72 days; circle) showed a linear relationship with light wavelength. Live body weight (30 days) = -0.154 × wavelength + 460.1 (*R* ^*2*^ = 0.8755), live body weight (72 days) = 0.164 × wavelength + 1081.2 (*R* ^*2*^ = 0.9251). Data are expressed as means ± SEMs (n = 4). Bars marked with different letters are significantly different from each other (*P* < 0.05).

Moreover, as shown in Fig 1, broilers treated with monochromatic light had faster growth rates than did broilers reared under broad-spectrum light. An increase in the relative cumulative growth rate was observed at 30 days of age in broilers reared under YL, GL and BL compared with the WL and FL groups. Moreover, YL caused a further improvement in growth rate between 60 and 72 days of age. In addition, a linear relationship was found between live body weight and light quality from the blue range through the red range (455 to 620 nm; Fig 2). At an early time point (30 days), wavelengths in the range of 455 to 620 nm were found to have a negative linear relationship with live body weight *(R* ^*2*^ = 0.876), with a decrease of approximately 15.3 g live body weight for each 100 nm (live body weight at 30 days = -0.154 × wavelength + 460.1). However, although broilers reared under YL were slightly heavier than broilers reared under RL (0.102%), a positive linear relationship was observed between wavelengths from 455 to 620 nm and live body weight in older chickens *(R* ^*2*^ = 0.9251), with an increase of approximately 16.4 g live body weight for each 100 nm (live body weight at 72 days = 0.164 × wavelength + 1081.2).

### Feed conversion

As broilers grew and developed, their feed consumption increased gradually. YL, GL and BL significantly improved feed efficiency compared with FL and RL treatments at both 45 and 60 days of age (*P* < 0.05; Table 1). Moreover, broilers reared under YL had greater feed efficiency than broilers reared under either GL or BL at these ages (*P* < 0.05). However, no differences in feed efficiency at 72 days of age were found among any of the groups (*P* > 0.05).

### Weights of drumstick

GL significantly increased tibia weight compared to the WL and FL groups (*P* < 0.05), but no significant treatment effect was observed among the RL, YL, BL, WL and CFL groups (Table 2). Similarly, GL significantly elevated thigh muscle weight compared to WL (*P* < 0.05), and no significant treatment effect was observed among the RL, YL, BL, WL and CFL groups (Table 2). The tibia and thigh muscle weights were similar percentages of the live body weight. The drumstick is generally regarded as a good indicator of skeletal development, which is related to the amount of weight a chicken broiler can carry. As expected, as shown in Table 3, the thigh weight of the drumstick was significantly (*P* < 0.05) and positively correlated with the tibia weight. Moreover, the thigh weight of the drumstick was significantly (*P* < 0.05) and positively correlated with the final body weight of broilers, which indicated that light quality can affect the growth of chicken broilers by increasing thigh weight.

**Table 2:** Weights of drumstick and viscera, and comb height of 78-day-old birds reared under different qualities of light. Each group of treated broilers was exposed to either WL (white LED), RL (red LED), YL (yellow LED), GL (green LED), BL (blue LED) or FL (fluorescent lamp). Data are expressed as means ± SEMs (n = 4).

Item	Light treatments groups					
	WL	RL	YL	GL	BL	FL
Tibia (g)	40.6 ± 2.7^b^	41.4 ± 2.0^ab^	42.0 ± 3.2^ab^	44.8 ± 3.9^a^	40.3 ± 4.3^b^	40.0 ± 0.8^b^
Thigh (g)	199.9 ± 10.2^b^	208.7 ± 18.0^ab^	217.3 ± 11.9^ab^	221.2 ± 7.7^a^	204.2 ± 7.4^b^	203.7 ± 16.8^b^
Heart (g)	3.9 ± 0.5^a^	4.0 ± 1.0^a^	4.0 ± 0.6^a^	3.5 ± 0.7^a^	3.8 ± 0.7^a^	3.6 ± 0.3^a^
Liver (g)	23.8 ± 2.1^a^	23.4 ± 1.7^a^	22.9 ± 1.9^a^	22.5 ± 3.5^a^	22.7 ± 2.6^a^	23.2 ± 2.5^a^
Stomach (g)	21.8 ± 3.2^a^	20.6 ± 2.1^a^	19.2 ± 1.6^a^	20.9 ± 2.5^a^	21.1 ± 2.4^a^	20.9 ± 3.5^a^
Comb (mm)	11.22 ± 1.84^b^	14.32 ± 2.32^a^	11.89 ± 2.14^a^	13.72 ± 2.17^a^	12.59 ± 1.92^a^	12.08 ± 2.33^a^

^ab^ Bars marked with different letters are significantly different from each other (*P* < 0.05).

**Table 3:** Correlation coefficients (r) between body weight and drumstick weight in broiler chickens slaughtered at 78 days of age^1^.

Correlations	Body weight	Tibia weight	Thigh weight
Body weight	1	0.295	0.568*
Tibia weight	0.295	1	0.553*
Thigh weight	0.568*	0.553*	1

^1^The correlation was investigated between two parameters, each of which was generated from the values obtained in six different lighting groups.

*P < 0.05.

### Weights of viscera

No significant difference was observed in weights of the viscera among all treatments (*P* > 0.05); no difference was observed in heart weight, liver weight or muscle stomach weight (Table 2). However, analysis only based on values without ANOVA indicated that: YL resulted in a greater heart weight values than that of broilers in the WL group by 2.5%, in the BL group by 5.0%, in the CFL group by 10% and in the GL group by 12.5%. WL and CFL resulted in higher liver weights than did YL (3.8% and 1.3%, respectively), BL (4.8% and 2.2%, respectively) and GL (5.8% and 3.1%, respectively), whereas chickens reared under GL and BL achieved greater muscle stomach weights than did those reared in RL (1.4% and 2.4%, respectively) or YL (8.1% and 9.9%, respectively).

### Comb growth

Light quality significantly influenced comb height, as shown in Table 2. Broilers reared under monochromatic light (RL, YL, GL and BL) had significantly greater comb heights than did broilers reared under broad-spectrum light (WL; *P* < 0.05; Table 2). Moreover, chickens treated with RL had the highest comb height among all of the treatment groups. The growth of the comb among broilers exposed to WL was inhibited considerably (Table 2).

## Discussion

Poultry rely heavily on visual cues when judging what is safe to eat and drink [37], and appropriate feeding behavior is facilitated by an innate predisposition to peck at small particles and flat shiny surfaces [38, 39]. Moreover, vision is probably the dominant sense in domestic poultry with respect to the majority of their behavior is mediated by vision. The results of the current study indicate that growth and development in broiler chickens are particularly affected by the light quality under which they are reared. In the present study, monochromatic light (455 to 620 nm) emitted from LED strips, especially human-friendly monochromatic yellow light, stimulated broiler growth compared to broad-spectrum light (WL and FL), as shown via measurements of live body weights, growth rates or feed efficiency. Thus, broilers reared under YL obtained the similar growth parameters with the broilers reared under GL and BL. Even, YL significantly improved feeding efficiency compared with GL and BL at 45 and 60 days of age. In addition, we observed that shorter-wavelength light (BL and GL) promoted growth during the early stages of development, whereas longer-wavelength light (YL) enhanced growth during the later stages of development.

As stated in the Introduction section, the results of studies of the effects of different light spectra on broiler growth have been inconsistent. The differences between these results can most likely be attributed to variability in the light sources, the light schedules, or the animal species studied or to a confounding effect of light quality. Thus, monochromatic LED light sources of specific wavelengths were used in the present study to eliminate potential of variability in the results of previous studies. In the present study, we found a quantitative relationship between live body weight and light quality (wavelength) between 455 and 620 nm. The wavelength of light (455 to 620 nm) was found to be negatively related (*R* ^*2*^ = 0.876) to live body weight at an early stage of development, with a decrease of approximately 15.4 g live body weight for each 100 nm, whereas the wavelength of light (455 to 620 nm) was found to be positively related to live body weight (*R* ^*2*^ = 0.925) in older chickens, with an increase of approximately 16.4 g live body weight for each 100 nm change in the wavelength of light.

Taken together, the similarity in feed intake across the specific monochromatic LED light sources and the adverse effects of WL and FL on broiler growth suggest that feed conversion was more efficient in broilers exposed to YL, GL or BL. A previous study showed that while feed consumption by broilers was highest from feeders illuminated by green light compared with those illuminated by red or orange light, there were no differences among rooms when a single colored light was used within each room [40]. Behavioral tests have suggested that broilers reared in red or white light are more active, as indicated by more aggression activity than reared under white light and red light [41]. The behavioral responses to red and white light may therefore contribute to the inferior growth observed in broilers exposed to longer wavelengths of light compared with wavelengths shorter than 580 nm (YL, GL and BL). Moreover, broilers have been shown to prefer green or blue light to red or white light, and they are calmer under green and blue light, as well [41]. In addition, blue light could be used to reduce the activity levels of broilers [42]. Taken together, these results may explain the improved feeding efficiency observed in broilers reared under shorter wavelengths in this study.

No significant difference was observed in organ weights among all treatments, either in heart weight, liver weight or muscle stomach weight, suggesting that endocrine functions were minimally sensitive to light quality. In addition to the gonads, the growth of many organs, including the comb, which is a secondary sex characteristic, is highly regulated by sex hormones [43, 44]. Comb height was measured in this study as an indicator of chicken development. The present study suggested that monochromatic light increased comb growth and that broad-spectrum light inhibited comb growth. Moreover, RL significantly increased comb height compared with WL. This finding may be due to variation in the penetration efficiency of light at different wavelengths. For example, longer-wavelength light has been shown to reach the hypothalamus more easily than has shorter-wavelength light in ducks [45] and quail [46]. As a result, longer-wavelength light had a maximal effect on gonadal development [47] and thus on comb growth. However, it would be important to elucidate more fully the processes whereby photons are converted to neural signals by photochemical changes in and outside the retina, as many biological processes, such as growth, may depend on the functions of retinal or extra-retinal photoreceptors.

In conclusion, the present study demonstrates that broilers exposed to monochromatic LEDs light (455 to 620 nm) exhibited enhanced growth and development compared with broilers reared under broad-spectrum light (WL and FL). Moreover, our results demonstrated that yellow light significantly improved growth performance in broilers. First, monochromatic yellow light (580 nm) improved measures of broiler growth performance, including live body weight, growth rate and feed conversion rate, compared with light from white LEDs or from fluorescent lamps. In addition, broilers treated with yellow light obtained the similar growth parameters with the broilers reared under green and blue light. Monochromatic green or blue light has been reported to stimulate the growth compared with those reared under white light in both a previous study [12]. Many reports have confirmed that green and blue light may have negative effects on humans [26, 30, 34, 48]. The use of monochromatic yellow LED light, which is very acceptable to humans, in poultry production could effectively prevent the negative effects of green or blue light on husbandry workers. Thus, due to concern for the welfare of poultry husbandry workers, we suggest that yellow LED lights can be applied to the broilers production. In addition, because the effects of the wavelength of light on the growth of broilers were observed to be age-dependent, altering the light environment at different ages may result in additional improvement in broiler growth; e.g., broilers could be exposed to shorter wavelengths (blue and green) at early stages and to longer wavelengths (yellow and red light) at later stages. However, the effects of changes in the light environment on broiler growth remain to be determined and more investigations are still needed.

## References

[1] Wells R. A comparison of red and white light and high and low dietary protein regimes for growing pullets. Br Poult Sci. 1971;12(3):313–25. 5142745 10.1080/00071667108415887

[2] Wabeck C, Skoglund W. Influence of radiant energy from fluorescent light sources on growth, mortality, and feed conversion of broilers. Poult Sci. 1974;53(6):2055–9. 4462103 10.3382/ps.0532055

[3] Coleman M, McDaniel G, Neeley W, Ivey W. Physical comparisons of lighted incubation in avian eggs. Poult Sci. 1977;56(5):1421–5.

[4] Proudfoot F, Sefton A. Feed texture and light treatment effects on the performance of chicken broilers. Poult Sci. 1978;57(2):408–16.

[5] Levenick C, Leighton A. Effects of photoperiod and filtered light on growth, reproduction, and mating behavior of turkeys. 1. Growth performance of two lines of males and females. Poult Sci. 1988;67(11):1505–13. 3237571 10.3382/ps.0671505

[6] Lauber J, McGinnis J, editors. Spectral sensitivity of avian growth and reproduction processes. Fed Proc; 1961.

[7] Ookawa T. Effects of bilateral optic enucleation on body growth and gonad in young male chicks. Poult Sci. 1970;49(1):333–4. 5440096 10.3382/ps.0490333

[8] Foss D, Carew L, Arnold E. Physiological development of cockerels as influenced by selected wavelengths of environmental light. Poult Sci. 1972;51(6):1922–7. 4660976 10.3382/ps.0511922

[9] Woodard A, Moore J, Wilson W. Effect of wave length of light on growth and reproduction in Japanese quail (Coturnix coturnix japonica). Poult Sci. 1969;48(1):118–23. 5355478 10.3382/ps.0480118

[10] Tamimie H. Light exposure of incubating eggs and its influence on the growth of chicks—I. Brooding chicks under different light regimens. Comp Biochem Phys A. 1967;21(1):59–63. 10.1016/0010-406x(67)90114-4 6033843

[11] Lauber JK. Photoacceleration of avian embryogenesis. Comp Biochem Phys A. 1975;51(4):903–7. 10.1016/0300-9629(75)90073-0 237716

[12] Rozenboim I, Robinzon B, Rosenstrauch A. Effect of light source and regimen on growing broilers. Br Poult Sci. 1999;40(4):452–7. 10579401 10.1080/00071669987197

[13] Charles R, Robinson F, Hardin R, Yu M, Feddes J, Classen H. Growth, body composition, and plasma androgen concentration of male broiler chickens subjected to different regimens of photoperiod and light intensity. Poult Sci. 1992;71(10):1595–605. 1454677 10.3382/ps.0711595

[14] Blatchford R, Klasing K, Shivaprasad H, Wakenell P, Archer G, Mench J. The effect of light intensity on the behavior, eye and leg health, and immune function of broiler chickens. Poult Sci. 2009;88(1):20–8. 10.3382/ps.2008-00177 19096052

[15] Buyse J, Simons P, Boshouwers F, Decuypere E. Effect of intermittent lighting, light intensity and source on the performance and welfare of broilers. World's Poult Sci Journal. 1996;52(2):121–30.

[16] Olanrewaju H, Thaxton J, Dozier W III, Purswell J, Roush W, Branton S. A review of lighting programs for broiler production. Int J Poult Sci. 2006;5(4):301–8.

[17] Karakaya M, Parlat S, Yilmaz M, Yildirim I, Ozalp B. Growth performance and quality properties of meat from broiler chickens reared under different monochromatic light sources. Br Poult Sci. 2009;50(1):76–82. 10.1080/00071660802629571 19234932

[18] Pan J, Yang Y, Yang B, Yu Y. Artificial Polychromatic Light Affects Growth and Physiology in Chicks. PloS one. 2014;9(12):e113595 10.1371/journal.pone.0113595 25469877 PMC4254831

[19] Falchi F, Cinzano P, Elvidge CD, Keith DM, Haim A. Limiting the impact of light pollution on human health, environment and stellar visibility. J Environ Manage. 2011;92(10):2714–22. 10.1016/j.jenvman.2011.06.029 21745709

[20] Chen L, Zhang XW. Which lamp will be optimum to eye? Incandescent, fluorescent or LED etc. Int J Ophthalmol-Chi. 2014;7(1):163–8. 10.3980/j.issn.2222-3959.2014.01.30 PMC3949479 24634884

[21] Lougheed T. Hidden Blue Hazard? LED Lighting and Retinal Damage in Rats. Environ Health Persp 2014;122(3):A81. 10.1289/ehp.122-A81 PMC3948029 24583823

[22] Lockley SW, Evans EE, Scheer F, Brainard GC, Czeisler CA, Aeschbach D. Short-wavelength sensitivity for the direct effects of light on alertness, vigilance, and the waking electroencephalogram in humans. Sleep. 2006;29(2):161–8. 16494083

[23] Dijk DJ, Archer SN. Light, Sleep, and Circadian Rhythms: Together Again. Plos Biol. 2009;7(6). 10.1371/journal.pbio.1000145 PMC2691600 19547745

[24] Lockley SW, Brainard GC, Czeisler CA. High sensitivity of the human circadian melatonin rhythm to resetting by short wavelength light. J Clin Endocrinol Metab. 2003;88(9):4502–5. 12970330 10.1210/jc.2003-030570

[25] Chellappa SL, Steiner R, Blattner P, Oelhafen P, Gotz T, Cajochen C. Non-Visual Effects of Light on Melatonin, Alertness and Cognitive Performance: Can Blue-Enriched Light Keep Us Alert? Plos One. 2011;6(1). 10.1371/journal.pone.0016429 PMC3027693 21298068

[26] Chellappa SL, Steiner R, Blattner P, Oelhafen P, Götz T, Cajochen C. Non-visual effects of light on melatonin, alertness and cognitive performance: can blue-enriched light keep us alert? PloS one. 2011;6(1):e16429 10.1371/journal.pone.0016429 21298068 PMC3027693

[27] Cajochen C, Munch M, Kobialka S, Kräuchi K, Steiner R, Oelhafen P, et al High sensitivity of human melatonin, alertness, thermoregulation, and heart rate to short wavelength light. J Clin Endocrinol Metab. 2005;90(3):1311–6. 15585546 10.1210/jc.2004-0957

[28] Lockley SW, Evans EE, Scheer F, Brainard GC, Czeisler CA, Aeschbach D. Short-wavelength sensitivity for the direct effects of light on alertness, vigilance, and the waking electroencephalogram in humans. Sleep. 2006;29(2):161 16494083

[29] Perrin F, Peigneux P, Fuchs S, Verhaeghe S, Laureys S, Middleton B, et al Nonvisual responses to light exposure in the human brain during the circadian night. Curr Biol. 2004;14(20):1842–6. 15498492 10.1016/j.cub.2004.09.082

[30] Vandewalle G, Schmidt C, Albouy G, Sterpenich V, Darsaud A, Rauchs G, et al Brain responses to violet, blue, and green monochromatic light exposures in humans: prominent role of blue light and the brainstem. PLoS One. 2007;2(11):e1247 18043754 10.1371/journal.pone.0001247 PMC2082413

[31] Vandewalle G, Schwartz S, Grandjean D, Wuillaume C, Balteau E, Degueldre C, et al Spectral quality of light modulates emotional brain responses in humans. P Natl Acad Sci USA. 2010;107(45):19549–54. 10.1073/pnas.1010180107 PMC2984196 20974959

[32] Papamichael C, Skene DJ, Revell VL. Human nonvisual responses to simultaneous presentation of blue and red monochromatic light. J Biol Rhythm. 2012;27(1):70–8. 10.1177/0748730411431447 22306975

[33] Wyszecki G, Stiles WS. Color science: Wiley New York; 1982.

[34] Van Der Lely S, Frey S, Garbazza C, Wirz-Justice A, Jenni OG, Steiner R, et al Blue blocker glasses as a countermeasure for alerting effects of evening light-emitting diode screen exposure in male teenagers. J Adolescent Health. 2015;56(1):113–9. 10.1016/j.jadohealth.2014.08.002 25287985

[35] Deep A. Impact of light intensity on broiler live production, processing characteristics, behaviour and welfare. College of Graduate Studies and Research in Partial Fulfillment of the Requirements for the Degree of Master of Science in the Department of Animal and Poultry Science, University of Saskatchewan, Saskatoon, 2010.

[36] Pallant J. SPSS survival manual: A step by step guide to data analysis using SPSS: McGraw-Hill International; 2010.

[37] Marples NM, Roper TJ. Effects of novel colour and smell on the response of naive chicks towards food and water. Anim Behav. 1996;51:1417–24.

[38] Appleby MC, Mench JA, Hughes BO. Poultry behaviour and welfare: CABI Publishing; 2004.

[39] Hogan JA. Development of food recognition in young chicks: I. Maturation and nutrition. J Comp Physiol. 1973;83(3):355. 10.1037/h0034669 4715301

[40] Smith L, Phillips R. Influence of colored neon lights on feed consumption in poults. Poultry Sci. 1959;38:1248.

[41] Prayitno D, Phillips C, Omed H. The effects of color of lighting on the behavior and production of meat chickens. Poult Sci. 1997;76(3):452–7. 9068043 10.1093/ps/76.3.452

[42] Rodenboog H, Noord, Oost P. Sodium, green, blue, cool or warm-white light. World Poultry. 2001;17(12):22–3.

[43] Balthazart J, Hendrick J. Steroidal control of plasma luteinizing hormone, comb growth and sexual behaviour in male chicks. J Endocrinol. 1978;77(1):149–50. 641433 10.1677/joe.0.0770149

[44] Zeller F. The effects of testosterone, dihydrotestosterone and oestradiol on the comb and oviduct of the female domestic fowl. J Reprod Fertil. 1973;34(1):147–8. 4719806 10.1530/jrf.0.0340147

[45] Benoit J. The role of the eye and of the hypothalamus in the photostimulation of gonads in the duck. Ann NY Acad Sci 1964;117(1):204–15. 14196641 10.1111/j.1749-6632.1964.tb48175.x

[46] Foster R, Follett B. The involvement of a rhodopsin-like photopigment in the photoperiodic response of the Japanese quail. J Comp Physiolo A. 1985;157(4):519–28.

[47] Pyrzak R, Siopes T. The effect of light color on egg quality of turkey hens in cages. Poult Sci. 1986;65(7):1262–7.

[48] Chang A M, Aeschbach D, Duffy JF, Czeisler CA. Evening use of light-emitting eReaders negatively affects sleep, circadian timing, and next morning alertness. P Natl Acad Sci USA. 2014: 1418490112. 10.1073/pnas.1418490112 PMC4313820 25535358

